# No evidence synthesis about me without me: Involving young people in the conduct and dissemination of a complex evidence synthesis

**DOI:** 10.1111/hex.13078

**Published:** 2020-06-08

**Authors:** Erin Walker, Elizabeth Shaw, Michael Nunns, Darren Moore, Jo Thompson Coon

**Affiliations:** ^1^ Great Ormond Street Hospital for Children NHS Foundation Trust London UK; ^2^ University of Exeter Medical School University of Exeter Exeter UK; ^3^ Graduate School of Education University of Exeter Exeter UK

**Keywords:** children, engagement, evidence synthesis, involvement, long‐term conditions, mental health, systematic review

## Abstract

**Objectives:**

To describe and reflect on the methods and influence of involvement of young people with lived experience within a complex evidence synthesis.

**Study design and setting:**

Linked syntheses of quantitative and qualitative systematic reviews of evidence about interventions to improve the mental health of children and young people (CYP) with long‐term physical conditions (LTCs).

**Methods:**

Involvement was led by an experienced patient and public involvement in research lead. Young people with long‐term physical conditions and mental health issues were invited to join a study‐specific Children and Young People's Advisory Group (CYPAG). The CYPAG met face to face on four occasions during the project with individuals continuing to contribute to dissemination following report submission.

**Results:**

Eight young people joined the CYPAG. Their views and experiences informed (a) a systematic review evaluating the effectiveness of interventions intended to improve the mental health of CYP with LTCs, (b) a systematic review exploring the experiences of interventions intended to improve the mental well‐being of CYP with LTCs and (c) an overarching synthesis. The CYPAG greatly contributed to the team's understanding and appreciation of the wider context of the research. The young people found the experience of involvement empowering and felt they would use the knowledge they had gained about the research process in the future.

**Conclusion:**

Creating an environment that enabled meaningful engagement between the research team and the CYPAG had a beneficial influence on the young people themselves, as well as on the review process and the interpretation, presentation and dissemination of findings.

## 1 BACKGROUND

Patient and public involvement and engagement (PPIE) in research is becoming increasingly recognized as good practice, especially within the UK. The National Institute for Health Research (NIHR) recently partnered with the other three nations of the UK to produce a set of Public Involvement Standards.^1^ further underlining their commitment to involving patients and the public in research. Benefits of incorporating PPIE into systematic reviews include increased relevance and timeliness, reduction in bias, increased accountability of the research process, better dissemination including greater accessibility and increased acceptance of the review results.^2^, ^3^ Furthermore, involvement in health research can support families and patients to feel more empowered, confident and enthusiastic, and allow children to develop skills they may have missed the opportunity to develop due to absence from school due to undergoing treatments for their conditions.^4^

There are various opportunities for PPIE within the systematic review process, including topic identification and prioritization; protocol development; review conduct; and knowledge translation and dissemination.^2^ A recent scoping exercise evaluating the conduct of 36 systematic reviews highlighted the level of benefit within the review process was proportionate to the level of PPIE time and resources; however, the level and type of PPIE conducted were inconsistent, with little record of the impact this involvement had on the review process.^3^

Challenges to involvement in systematic reviews are having sufficient time and resources, distinguishing the appropriate people to involve, and concerns that involvement could have a negative impact on scientific rigour.^2^ They can also include negative beliefs and attitudes about PPIE; lack of knowledge about, and skills in, PPIE; dysfunction and hierarchies on the staff side; and uncertainty over how to resolve differences between health‐care professionals and involved people.^5^ These challenges risk public engagement being undertaken in a surface‐level manner, as initially described within Arstein's (1969) ‘ladder of citizen participation’ which outlines the eight various levels of public involvement, beginning with the lowest levels of ‘Manipulation’ and ‘Therapy’.^6^ The ‘Ladder of Engagement and Participation’ stipulates that public engagement can occur at several levels ranging from the provision of Information and progressing through Consulting, Involving, Collaborating and Devolving—whereby the decision making is passed entirely to individuals and the community.^7^ If PPIE is to be more than a tokenistic approach to facilitate the acquisition of funding, or validate researchers’ own opinions, and instead of truly involving members of the public in an equal collaboration, the barriers to conducting PPIE need to be overcome. Some potential solutions have already been discussed, including finding people who have relevant expertise by experience, interest and availability, involving them in meaningful tasks throughout a study, and keeping them engaged through the provision of dedicated and experienced support.^8^, ^9^ This latter point may involve the joint training of patients and health‐care professionals; formalizing patient roles with role descriptions; informal interactions to build trust and rapport; involving patients from the very beginning of a project; small team size; frequent meetings; and active solicitation of patient input during meetings.^5^ However, finding widespread, easily accessible guidance to overcome challenges such as these is not always straightforward. PPIE methods are not routinely taught on traditional research methods courses, and whilst some journals now request authors describe PPI in manuscripts for submission word count, restrictions often preclude the full reporting of PPIE methods within journal articles.^10^ While a small literature is growing on systematic reviews of PPI with various patient groups, there is much less practical advice available on the planning and delivery of meaningful PPIE in evidence synthesis. This is even more so in research on children and young people. Gierisch et al have proposed PPIE practices for future evidence syntheses to consider. These include careful selection of individuals to involve; collaboration on goals of the PPIE, roles of the involved people and expectations for areas of influence in the evidence synthesis; research training by researchers so that involved people can effectively contribute; allocating sufficient resources (including time and finances); and on‐going reflection of the PPIE to ensure it is still beneficial to all parties and the research.^11^ However, it can still be difficult for researchers to know how to practically implement such suggestions whilst undertaking a systematic review.

Given our experiences in the area, and genuinely believing that it would add value and help root our research in the needs and experiences of children and young people (CYP), we planned a comprehensive programme of PPIE throughout a recently conducted NIHR‐funded evidence synthesis.^12^ This evidence synthesis entailed a qualitative systematic review, a quantitative systematic review and an overarching synthesis on interventions intending to improve the mental health of CYP living with long‐term physical conditions. This article is intended to describe, from the perspective of the research team, the process of engaging and working alongside CYP with long‐term physical health conditions and mental health difficulties and their parents whilst conducting and disseminating a complex evidence synthesis. These descriptions emerged from reflections and open discussions that took place during the conduct of the review and later during preparation of this manuscript. We also aim to highlight the influence this involvement had on each stage of the project, the research team and the young people themselves. We hope this paper provides an example of how to overcome some of the challenges of incorporating PPIE within systematic reviews and implement some of the suggestions made by existing research.

### 1.1 Language

We recognize there are many terms used to describe people who have experience of illnesses like diabetes, anxiety, rare diseases or a condition that requires them to access health services, such as broken limbs or pregnancy, including patient, service user and consumer. We use the term ‘patient’ to describe these individuals. ‘Public’ refers in this case mostly to parents and carers, or people interested in the area of the intersection of mental health and physical long‐term conditions for CYP.

## 2 METHODS

Our approach to involving CYP in this research was guided by our past experiences in the area^13,14^ as well as the values and principles framework set out by INVOLVE^15^ of respect, support, transparency, responsiveness, diversity and accountability, as highlighted by the examples provided in Table 1 and explained in detail below. The UK Standards for Public Involvement had not been published at the time we undertook this work.

**Table 1:** Descriptive examples of how we engaged with INVOLVE values and principles

INVOLVE principles and values	Descriptive examples
Respect	Researchers valued the unique contribution that the CYP and their parents could make to the project. During facilitated discussions, the dedicated PPIE co‐applicant (EW) used her skills and experience to ensure that all members of the group had opportunity to contribute and that their contribution was acknowledged by the group. One of the parents was a co‐applicant on the funding application and the main report. CYP and their parents co‐wrote the plain language summary in the main report which was subsequently used as a basis for newsletter articles. They also contributed a prologue and an epilogue. Members of the group recorded podcasts and some of the CYP presented findings from the project at two conferences. CYP and their parents said they valued the knowledge and experience of the researchers and reported that they enjoyed learning about the evidence behind some of the treatments they had received. Each parent and CYP were provided with a gift voucher for every meeting they attended in recognition of their time and experience.
Support	Our dedicated PPIE co‐applicant provided support on an on‐going basis throughout the project both to the researchers and the CYP and their parents. She provided expert facilitation during discussions and ensured that all attendees at the meetings were comfortable and secure in their roles. She also took the time to directly contact CYP and/or their parents over email or telephone after each meeting to ensure they were not negatively affected by the topics discussed and remained contactable outside schedule meetings. Plans for the PPIE were developed with the PPIE co‐applicant who ensured that the timelines and resources provided were adequate. Researchers worked together to make sure that plans for involvement remained feasible throughout. Meetings were held at a familiar location (for the CYP and their parents) at a time that suited them best; travel expenses were reimbursed in cash on the day. The PPIE coordinator supported the making of transport arrangements where necessary.
Transparency	When the CYP were asked to join the group, we outline our plans for involvement, the time commitment involved, our plans for the project, why their views and experiences were important to us and the project, how we would capture their input and the extent to which it may be possible to influence the project and/or future health care. Researchers spent time at each meeting describing the purpose of the meeting, explaining how their input from previous meetings had influenced the project and outlining the timetable for the project and the next steps.
Responsiveness	At each meeting, researchers actively responded to suggestions made by the CYP and their parents. Where we could not incorporate suggestions, we were transparent and open and explained the rationale behind our decision.
Fairness of opportunity	Researchers made sure that CYP and their parents understood that if they could not attend a meeting in person, this would not exclude them from attending future meetings. If CYP were unable to attend meetings due to time conflicts or poor mental or physical health, EW sent them a summary of what was discussed and invited them to give their own feedback over the telephone or via email. Researchers approached attendance at meetings flexibly, knowing that not all of the CYP would be able to attend every meeting, and we regularly reminded the CYP that they were under no pressure to attend as their involvement was voluntary, and we were sensitive to their health conditions. Where opportunities arose for the CYP to present the work at conferences, we ensured that all members of the group were given a fair chance to attend with appropriate support provided where necessary.
Accountability	At the end of the project, we all spent time discussing and actively reflecting on the PPIE experience. CYP and their parents were given the opportunity to read and comment on the final report including the researcher's report of the involvement activities. CYP and their parents were involved in planning the dissemination activities to ensure that the research would be available to other CYP and their parents.

### 2.1 Context

When forming the PPIE group, we took care to consider issues which may have influenced young people's desire and ability to participate. Group meetings were held at Great Ormond Street Hospital for Children NHS Foundation Trust (GOSH) and facilitated by EW, who had years of experience involving CYP in health research. The evidence synthesis researchers from Exeter University (JTC, LS, DM and MN) also attended each meeting. The hospital was already known to CYP, although the Clinical Research Facility, where meetings were held, was not, which is why we picked that space to meet. The research team made concerted efforts to reduce any anxiety surrounding attending. We discussed and agreed our general ‘rules of engagement’ at the beginning of our first meeting. We were clear with the young people that they did not have to participate and could withdraw their involvement at any time. We also reassured CYP that they did not have to talk about their own health experiences, or topics they felt uncomfortable with and did not have to answer questions they did not want to. We also reiterated in all meetings that we were creating a ‘safe space’ and that CYP and researchers were free to discuss any issues in confidence. For meetings 3 and 4, which both CYP and their parents attended, time was spent both all together in one group and in separate groups (CYP and parents) to enable free and honest discussion without the fear of disclosure in front of one's parent/child. If CYP were unable to attend meetings due to time conflicts or poor mental or physical health, EW sent them a summary of what was discussed and invited them to give their own feedback over the telephone or via email. We made it clear that being unable to attend a meeting in person would not exclude CYP from attending future meetings, as we understood that their health experiences could vary across the 15‐month project. All travel expenses were reimbursed in cash on the day of the meeting, and CYP were given high‐street vouchers for each meeting they attended in recognition of their time and expertise.

### 2.2 Approach

One of the co‐investigators of the larger project in which this PPIE work was embedded, a consultant psychiatrist in a complex epilepsy service at GOSH, identified and approached four CYP who had experience of neurological conditions and mental health problems. With approval of the CYP, the PPIE co‐investigator (EW) then contacted these individuals about getting involved in the study. Similarly, a PPIE colleague of EW who undertook the involvement in a joint adolescent rheumatology service run by GOSH and University College London Hospitals identified and approached CYP with rheumatological conditions and mental health problems too for involvement in this study. Where permitted, EW followed up to seek their involvement. All CYP approached agreed to become involved.

The ethos behind PPIE in research was first explained to the CYP and parents via email. No signed consent was obtained, as we regarded the CYP as equal partners in the research process, rather than research participants.^16^

All members of the research team worked proactively to ensure that the CYP and parents involved with the project were respected, and this was reciprocated by them in turn. For example, at the beginning of the project, we outlined our plans for involvement, why their views and experiences were important to us and the project and how we would capture their input. We regularly reiterated that they were the only individuals in the team able to provide a perspective on what it is like to be a young person dealing with a long‐term physical condition and mental health issues and their experiences were invaluable to our combined success, since that expertise was critical to the research. We allowed plenty of time for questions and discussion and were transparent about the extent to which they would be able to influence the research, given that this was a funded project with clearly laid out objectives, methods and timelines. During facilitated discussions, the dedicated PPIE co‐applicant (EW) used her skills and experience to ensure that all members of the group had opportunity to contribute and that their contribution was acknowledged by the group. We spent time at each meeting describing the purpose of meetings, explaining how their input from previous meetings had influenced the project, outlining the timetable for the project and the next steps and actively responding to suggestions made by the CYP and their parents. Where we could not incorporate suggestions, we were transparent and open and provided a justification for our decision. We also communicated directly with the CYP, and kept parents informed, except where some CYP preferred their parents be the primary communicators.

We unequivocally support fairness of opportunity and made an effort to involve CYP without discrimination. For example, we approached attendance at meetings flexibly, knowing that not all of the CYP would be able to attend every meeting, and we regularly reminded the CYP that they were under no pressure to attend as their involvement was voluntary, and we were sensitive to their health conditions. CYP did not have to contribute to every meeting in order to remain involved with the project. Where opportunities arose for the CYP to present our work at conferences, we ensured that all members of the group were given a fair chance to attend with appropriate support from parents and the PPIE coordinator provided where necessary. We firmly believe that we are accountable to those affected by the research we conduct, and this inspires us to conduct research to the best of our abilities.

### 2.3 Involved young people

A group of eight CYP between 10 and 17 years of age, including 3 male, indicated they would like to be involved with the project. These CYP lived with primarily neurological or rheumatic LTCs (although most had other comorbid physical conditions), and all had experienced issues which affected their mental health and emotional well‐being. Five parents attended the two final face‐to‐face meetings.

### 2.4 Procedure

CYP were involved seven times during the project, including four face‐to‐face meetings whilst the 15‐month evidence synthesis was being conducted (January 2016‐April 2017), and then during three dissemination activities after the study had closed. All four meetings conducted during the course of the project took place at GOSH as this was a place familiar to CYP and their parents with good transport links. Meetings were scheduled at timepoints in the research where involvement would be most appropriate, to best meet the needs of the research and the CYP. Although the gaps between some meetings were quite long, more frequent meetings or meetings scheduled at a different frequency would not have been useful and could have placed additional burden on CYP and their parents. Proposed meeting dates were always shared in advance with CYP and their parents. After each meeting, the PPIE co‐investigator (EW) checked in with the CYP via email.

We did not originally intend to involve parents. However, part way through the study, it was observed that the parents had formed a spontaneous group whilst the researchers and CYP met and were sharing stories of their children's experiences in accessing physical and mental health care. We felt that their views would provide a valuable perspective to the project and decided to invite parents to attend the final two face‐to‐face meetings.

We discussed potential dissemination options throughout the project, exploring how and where individuals sought information about their health and possible treatments for ill health. As a result of these discussions, we undertook five activities with the CYP and parents: (a) two podcasts—one to communicate the findings and one to share experiences of being involved in the project; (b) three conference presentations—one oral presentation and one poster presentation at the INVOLVE 2017 meeting, and one oral presentation at the Cochrane Colloquium 2018; (c) a blog post; (d) contributions to the final report; and v) a plain English summary for distribution to interested organizations.

Unless otherwise stated, the reflections provided in this paper are those of the research team. Permission to reproduce text from emails was provided by the CYP.

## 3 RESULTS

Within this section we describe our PPIE activities as well as detailing the influence each activity had on the project. A summary of this information is provided in Table 2.

**Table 2:** Summary of activity at each meeting

Who?	What did we do?	Influence on project
Meeting 1—Month 2—London Aim: To introduce the project and the team members, set out plans and expectations, and develop rapport		
Researchers (n = 5) and CYP (n = 7)	Ice‐breaker activity to help members of the team get to know one another. Presentations: Care was taken to ensure language was accessible to CYP. Facilitated discussions to address: What do CYP think about the focus of the project? How do CYP define ‘mental health’ and ‘long‐term conditions’?	Provided a ‘sense‐check’ for the researchers. Provided reassurance that our search strategy was capturing all the key terms. Reinforced the importance of acknowledging the link between physical and mental health and how interventions aimed at treating one aspect of mental health may impact on another. Strengthened our justification for including all outcomes in the synthesis of Review 1.
Meeting 2—Month 9—London Aim: To gain a better understanding of the young people's experience of different types of interventions and the outcomes used to measure their effectiveness		
Researchers (n = 5) and CYP (n = 8)	Facilitated discussion to catch up since the last meeting. Presentations to update progress on the reviews. Card sorting activities to rank interventions and outcomes. Introduction to the types of information within published papers that will contribute to Review 2 Facilitated discussions around activities to address: Which of the ways of treating mental health problems that we had identified did the CYP predict would work best and why? Which outcomes did the CYP think were important? Does the information in published papers resonate with the experiences of the CYP? How should we disseminate the findings?	Reinforced the importance of a wide range of outcomes and the impacts of interventions on relationships with friends and family members. CYP emphasized the importance of school attendance and coping with school. This discussion informed our implications for future research. As a group, we decided to consider a podcast as a dissemination activity involving the CYP and to include time for editing plain language summaries for different end users in the final meeting.
Meeting 3—Month 13—London Aim: To discuss the preliminary findings from the reviews and consider whether the researchers’ interpretation and presentation of them was in keeping with the CYP and their parents’ experience		
Researchers (n = 5), CYP (n = 5) and their parents (n = 5)	CYP and parents discussed the review findings in separate rooms. Presentation of preliminary findings to both groups: CYP created individual ‘spider maps’ of their perception of an ideal intervention for their mental health difficulties in response to questions from the researchers. Parents had an open discussion about what they felt would make a ‘good’ intervention. Facilitated discussions to address: What did the CYP and their parents think of the findings from the reviews, and the research team's interpretation of the overarching synthesis? Had the study team missed anything in their interpretation of the results? To what extent did the findings resonate with the experience of the CYP and their parents? How should we disseminate the findings? Discussion of next steps, mindful that the project was nearing conclusion.	Reinforced and challenged ideas in the preliminary synthesis of qualitative research. Informed the analysis, interpretation and presentation of the overarching synthesis. Strengthened both the content and final structure of the syntheses.
Meeting 4—Month 15—London Aim: To begin to co‐produce some of the project outputs.		
Researchers (n = 5), CYP (n = 7) and their parents (n = 5), podcast recording specialist (n = 1)	Presentation to update on progress. CYP and parents split into two rooms; in one room, researchers worked with the group to amend and edit a plain language summary, and in the other room, the podcast recording specialist worked with the group to record material for the podcasts and then the groups swapped so that both groups contributed to both activities. Facilitated discussions to address: How could we ensure the results from the research would reach CYP with long‐term conditions and mental health problems, and their families? Who else should see the findings of this research? How could we make sure the findings were understood?	Children and young people and their parents recorded material for two podcasts—the first discusses the findings of the project, the second their experiences of being involved. Children and young people and their parents also co‐wrote plain language summaries of the findings—one for the final report and adapted versions for their respective audiences (children and young people and parents).

### 3.1 Face‐to‐face meetings

#### 3.1.1 Meeting 1

The first face‐to‐face meeting was in February 2016, in month two of the project. This was the first time the researchers met with the CYP, and the CYP met with each other. Seven CYP attended the meeting with researchers (DM, JTC, LS, MN), which was facilitated by EW.

The aim of the meeting was to introduce the project and the research team, provide explanation of challenging concepts and manage expectations of the scope of the project, what it would involve and what it might achieve, but most importantly to develop rapport and relationships between the CYP and researchers, and within the CYP group itself. The research team gave a presentation to explain what a systematic review was, how one was carried out and the role of patients and the public within this. There was also a focus on the idea behind, and plans for, the research activity and the involvement work stream. Care was taken to ensure that the language used was age‐appropriate and that opportunity to ask questions was provided. An ice‐breaker activity allowed for the research team and CYP to get to know one another and make it easier for people to ask questions. Key questions for the research team to address in the meeting were as follows: What do CYP think about the focus of the project? And how do CYP define ‘mental health’ and ‘long‐term conditions’?

All the CYP were unanimously enthusiastic about, and supportive of, the research questions and aims of the project. They all spoke about how having a physical long‐term condition was inextricably linked to mental health, although to varying degrees. CYP voluntarily, and organically throughout the discussion, disclosed some of their own experiences and diagnoses of physical and mental ill health although this was not required for involvement.

#### 3.1.2 Influence on the project

CYP were asked about the terms they use to describe mental health with their peers, to check and make sure the researchers had not missed any key terms for the search strategies of the qualitative and quantitative systematic reviews. This meeting provided the opportunity for researchers to ‘sense‐check’ the proposed project. CYP felt that an intervention intended to improve one aspect of their well‐being may also influence other aspects; for example, an intervention aiming to improve anxiety symptoms may also make it easier for CYP to attend school. This reinforced the importance of evaluating all potential outcomes within the synthesis of quantitative evidence.

### 3.2 Meeting 2

The second meeting took place in month nine of the project; the quantitative and qualitative reviews had begun, and the research team had some early indication of the types of interventions in the included studies. The key focus for the meeting was to gain a better understanding of the young people's experiences of different types of interventions for treating their mental health problems and the outcomes used to measure the intervention's effectiveness in the included studies. Key questions we wished to address were as follows: Which of the ways of treating mental health problems that we had identified in the first review did the CYP predict would work best? What might be the reasons for this? Which outcomes that we could measure after people have received treatments or interventions did the CYP think were important?

Eight CYP attended alongside the research team. As a number of months had passed since the previous meeting, we initially spent time developing rapport with, and between, the group members before MN and LS provided updates on the two reviews, including how input from the CYP during the last meeting had influenced the project. We then undertook two card sorting activities designed to address our key questions and elicit conversation and reflection between the CYP and the research team. At the end of the meeting, the group started to discuss ways in which they would be interested with helping to disseminate study findings.

The first activity involved making sense of the different types of interventions that the reviews had revealed for treating mental health problems in CYP with long‐term conditions (eg cognitive behaviour therapy, play therapy, music therapy, relaxation). This encouraged the CYP to discuss their own experiences with accessing different types of treatment, and successes or failures they had with them. CYP were then provided with cards each showing the name of an intervention identified in Review 1 and asked to sort them according to how well they thought they would work. This gave the young people a way to draw upon their experiences with interventions and also to give ideas about experiences and barriers that would be important for the qualitative review.

The second activity focused on the different outcomes included in studies as targets for intervention (eg depression, anxiety, coping, pain, family relationships). The CYP were presented with cards showing common intervention outcomes from Review 1 and were asked to rank them in order of importance. They were also issued with blank cards to write down outcomes which they considered to be important and which had not been identified by Review 1. Some of the outcomes they added include fatigue and social functioning, and the group also discussed the interplay between the outcomes identified. CYP felt there was a lack of focus on school outcomes, which they considered to be important.

Finally, we discussed some of the quotes identified from Review 2. This activity served a number of purposes; firstly, it enabled the researchers to illustrate the type of information that would be included in review 2 and how it is presented in research papers, and secondly, it allowed the CYP to compare and contrast the experiences presented in qualitative research studies with their own. Finally, it helped the researchers to understand the degree to which the research and preliminary themes resonated with the CYP.

#### 3.2.1 Influence on the project

Discussions at this meeting underlined the importance of considering a wide range of outcomes in the reviews including elements like impacts on relationships with friends and family members. Much of the discussion was centred on school‐related outcomes, for example school attendance and coping with school. These outcomes were not frequently reported in the included studies, and this influenced our recommendations for future research. The researchers hoped the discussion around outcomes might facilitate the structure of the synthesis within the review of quantitative evidence. Unfortunately, due to the paucity of evidence identified for some of the interventions and gaps in the evidence relating to interventions and outcomes that the CYP found useful, this was not possible.

CYP were keen to be involved in creating a podcast and editing plain language summaries to share the project findings, and plans for conducting these activities were included in the project timeline.

### 3.3 Meeting 3

The third meeting of the group took place in month 13 of the project. This was the first meeting attended by the self‐convened parent group. The meeting was attended by five CYP and five parents in addition to the research team. Key questions to address in this meeting were as follows: What did the CYP and their parents think of the findings from the reviews, and the research team's interpretation of the overarching synthesis? And, had the study team missed anything in their interpretation of the results? To what extent did the findings resonate with the CYP and their parents?

As this was the first time the parents had joined the group and there had been 4 months since the previous meeting, the researchers spent time reviewing the aims of the project. The group separated, with the CYP in one room and parents in the adjacent room. This was so that all members of the group could disclose their opinions and potentially sensitive experiences honestly, without feeling they must inhibit themselves in front of their child/parent.

Two researchers (DM and JTC) facilitated the group of CYP, and two researchers (MN and ES) facilitated the activities with parents.

DM guided the CYP through a task where, using A3‐sized paper, they were asked to respond to questions about their preference for features, content and delivery of an intervention and in doing so create a spider diagram of their notion of an ideal intervention for CYP with long‐term conditions and mental health problems. Examples of the types of question we posed include ‘Where would your ideal intervention be delivered eg in hospital, at school, somewhere else?’ ‘Who would deliver it?’ ‘Would you be on your own or in a group?’ and ‘How often would you attend?’ The activity was based upon findings arising from the qualitative systematic review. We framed questions in terms of features the CYP would prefer in an intervention aimed at improving their mental health and also asked them to consider the reasons for their preferences. We then discussed the qualitative systematic review and overarching synthesis findings in relation to CYP responses. We chose to structure the activity in this way as we were keen to capture ideas and reflections that went beyond presenting findings and seeking agreement.

MN and ES presented the key findings from both reviews to the parents and provided an outline of the preliminary findings from the overarching synthesis. An open discussion was held regarding these results, and parents were asked about what they thought would make a good intervention.

We also discussed approaches to dissemination, and the parents expressed interest in helping to write a plain English summary of the results and recording material for podcasts.

The CYP and parents then rejoined to conclude the meeting, and we discussed next steps. Researchers were careful to manage expectations, mindful that the project was soon to finish.

#### 3.3.1 Influence on the project

The discussions provided support to the preliminary themes which were arising from the synthesis of qualitative research and the overarching synthesis.

For example, the CYP placed great importance on the expertise and ability of the therapist to create a safe environment with confidentiality and clear expectations being highly valued, alongside flexibility with delivery, venue and frequency of sessions. The parent group highlighted the need for interventions to involve systems around the child and the challenges in having the mental health needs of CYP recognized and met by schools and primary care clinicians.

Several points arose during both meetings which challenged the syntheses, and encouraged the researchers to return to the data and consider how these additional perspectives should be incorporated into the final findings. An example of this was the suggestion that mental health interventions should acknowledge that the needs of CYP were serious and therefore did not always need to contain an element of ‘fun’. Overall, the discussions with parents and CYP strengthened both the content and final structure of the syntheses conducted within the second and third components of the project.

### 3.4 Meeting 4

The fourth and final meeting was attended by the core PPIE research team, 7 CYP, 5 parents and a Science Communications Specialist. The aim of this meeting was to begin to co‐produce some of the project outputs. Key questions to address in this final meeting were how could we ensure the results from the research would reach CYP with long‐term conditions and mental health problems, and their families, who else should see the findings of this research and how could we make sure the findings were understood?

The meeting began with a reminder of the project and an update on progress with preparation of the final report to the funder. The group then split, with CYP in one room and parents in an adjacent room. DM and MN guided an activity with CYP to edit a pre‐prepared draft of the plain English summary shared using the computer and projector facilities. Using reviewing functionality in MS Word (ie Track Changes), the group went through the summary in great detail, editing each line of text. Words CYP considered to be jargon were debated for their meaning and replaced with a more accessible word where possible. The CYP offered suggestions for who should be given the plain English summary to read, and whether they thought it could help make a difference to their understanding or attitude towards CYP with long‐term physical conditions.

In an adjacent room, ES, JTC and the Science Communications Specialist worked with parents to record material for a podcast. They were asked about their experiences of involvement and their views on the research—its aims, the issues for CYP with mental health problems and long‐term conditions, and their experiences of parenting CYP in that position.

The two groups then swapped rooms, so all parents and young people were involved in both tasks.

#### 3.4.1 Influence on the project

CYP and their parents recorded audio for the podcast and helped to edit plain language summaries. They had strong views on the type of clinicians, educators and researchers the research findings should be shared with, which was incorporated into our dissemination strategy.

## 4 DISSEMINATION ACTIVITIES

Dissemination activities undertaken in collaboration with the CYP and parents involved in this project were as follows:

### 4.1 Two podcasts

One to communicate the findings and one to share experiences of being involved in the project.

The podcasts were made available on the project webpage and shared during conference presentations and on social media. On publication of the final report in May 2019, links to the podcasts were shared with relevant organizations alongside the Briefing Paper and in some cases formed the basis for newsletter updates, for example the Children and Young Peoples Mental Health Coalition and the MQ Research Round Up.

### 4.2 Three conference presentations

One oral presentation and one poster presentation at the INVOLVE 2017 meeting, and one oral presentation at the Cochrane Colloquium 2018.

As the preparation for these events took place after the work on the project was complete, the research team (MN, EW, JTC, DM, ES) prepared the conference abstracts. The abstract submitted to the INVOLVE 2017 conference was initially accepted for a poster presentation, and then, at short notice we were offered the opportunity to also give an oral presentation. The PPIE co‐investigator (EW) created the presentations with input from the CYP. Two of the young people agreed to co‐present at the conference and were fully involved in this process.

The abstract submitted to the 25th Cochrane Colloquium held in Edinburgh in September 2018 was accepted as an oral presentation. Whilst the abstract had been written by members of the research team, the presentation was co‐created and co‐delivered by the PPIE co‐investigator and one of the young people. During this conference, EW and the young person were invited to record material for the *Mental Elf* podcast series to talk about the findings and their experiences of being involved in the project.

### 4.3 A blog post

The PPIE co‐investigator and the two young people shared their reflections on presenting at the INVOLVE conference for a blog post which appeared on the research team's blog.

### 4.4 Contributions to the final report

Following the final face‐to‐face meeting, all the CYP and their parents were asked to contribute material via email to the final report, for a prologue (Foreword) and epilogue (Closing Messages).

### 4.5 Plain English summary for distribution to interested organizations

The co‐produced plain English summary was used in the final report. Material from this summary, the prologue and the epilogue were incorporated into the project webpage and also used in a Briefing Paper produced by the research team for dissemination to clinicians and commissioners.

### 4.6 Influence on project

By continuing our engagement with CYP and their parents through the dissemination stage of our project, we were able to ensure that the messages arising from our project that they felt were most important were communicated to the audiences they felt were most appropriate, via a variety of accessible media.

## 5 OVERALL REFLECTIONS ON THE IMPACT OF INVOLVING CYP AND THEIR PARENTS ON THE PROJECT

The contribution of the CYP and their parents enabled us to produce a robust evidence synthesis grounded in the experiences and insights of CYP and their parents. This is evidenced by the degree to which external stakeholders, end user groups and practitioners have identified with the presentation of the findings. An example of this is the widespread acknowledgement that considering mental and physical health separately is not helpful—a view that was strongly held by the CYP. Furthermore, our implications for future research were informed not only by existing evidence but also by gaps in the evidence identified by those with experience and insight of the key issues.

Throughout the course of the project, we discussed and reflected on the balance between burden and benefit for the CYP, their parents and the researchers.

## 6 OVERALL REFLECTIONS ON THE INFLUENCE OF THE CYP AND THEIR PARENTS ON THE RESEARCH TEAM

Although the PPIE activities required extensive planning and discussion and travel to London, they were not felt to be a burden by the research team. Time and resource for the activities (and their preparation) were built into the project timeline, and the meetings were always felt to be worthwhile in terms of project progress. The researchers found working alongside the CYP both humbling and inspiring, especially given the nature of their physical long‐term conditions and mental ill health experiences—life and activities of daily living were very hard for many of the CYP. These were bright, intelligent young people who had experienced significant adversity at young ages. It was motivating, and provoked continual reflection on the needs of the group and how to best engage and involve them in the research process in a way which was truly meaningful, and provided an additional sense of accountability to ensure that we conducted the review to the best of our ability.

## 7 REFLECTION ON THE INFLUENCE OF BEING INVOLVED IN THIS PROJECT ON THE CYP AND THEIR PARENTS

The young people said that in addition to finding their involvement in shaping the research project empowering, they also benefited simply from meeting other CYP with long‐term physical conditions and mental health issues. Furthermore, feedback received via email following the first meeting included:

It was good to hear other people's points of view. [CYPAG member 1]

I think it was really helpful having other people who have gone through the same things as you, that understand you. [CYPAG member 2]

Parents supported this observation, indicating that they felt it was important for their child to meet other people in similar circumstances to them, who were getting ‘on with their lives’.

## 8 DISCUSSION

In this paper, we describe how we approached involving CYP and their parents in the conduct and dissemination of a complex evidence synthesis of interventions to improve the mental health of CYP with physical long‐term conditions. We also describe our reflections on the influence of the involvement on the project, the research team and our involved people. Creating an environment that enabled meaningful engagement for both the research team and the CYP not only had a beneficial influence on the review process and the interpretation and presentation of the findings, but also for the parents and CYP themselves.

The influence of CYP PPIE within each stage of the evidence synthesis has already been outlined above. Overall, the involvement of CYP and their parents within this project has ensured that it remains grounded in the reality of the people it is intended to benefit, with the final product being shared with clinicians and commissioners, whose decisions shape the care these families receive.

In addition to influencing the final output from the project, involving CYP and their parents also had an impact on the researchers and the CYP and the parents themselves. Whilst we did not formally set out to evaluate the involvement, we were able to explore our mutual experiences during the recording of one of the podcasts. These discussions highlighted benefits for all the individuals involved, which went beyond producing a research report.

As researchers, we were acutely aware throughout the project of the need to ensure that the involvement was meaningful for everyone. Learning of additional, unanticipated benefits like increasing research knowledge and meeting others who are similar helped us feel more confident that the involvement had indeed been mutually meaningful and beneficial.

## 9 POTENTIAL CHALLENGES AND STRENGTHS

Planning for meaningful involvement of CYP within this research raised a number of challenges: (a) we needed to involve CYP and accommodate their physical and mental health needs, (b) we were asking CYP to talk about the potentially sensitive topics of their experiences of mental and physical ill health, (c) the project was a fully funded evidence synthesis with potentially limited opportunities for the involvement to have an impact on findings and (d) we had a defined budget to cover involvement activities, which would preclude major changes to approach.

A positive and fruitful working relationship between the CYP, their parents and the research team was fostered by the shared motivation to ensure that the outcomes from the project were useful to CYP living with mental illness and long‐term conditions. The research team deliberately sought opportunities to incorporate their views to shape the project's process, findings and outcomes. Accommodating the other demands on the time of the CYP and parents, as well as physical and mental health needs, took flexibility on the part of the research team and efforts from CYP and their parents. The incorporation of a dedicated PPIE co‐investigator was essential in facilitating flexible involvement. EW played a crucial role in maintaining contact with CYP between meetings, summarising meeting content for those who were unable to attend and supporting them to attend meetings where they could.

CYP were encouraged to contribute however they felt able to. One young woman who, due to the scheduling of an injection she would receive to manage her LTC, typically felt highly unwell on Saturdays, which was when our meetings occurred. She missed the first meeting as a result, but attended the subsequent three meetings and was able to participate via email; she also helped with dissemination. Another young man became increasingly busy with school due to examinations and was unable to attend the final two meetings. A couple of other young people had varying levels of health across the 15 months. Despite these challenges, each individual CYP contributed to this project, and allowing flexibility with attendance and method of involvement helped ensure the involvement was accessible to all of them.

Care was needed to ensure that discussing their experiences did not cause the mental or physical health of CYP to worsen or mean that attending meetings was experienced as similar to attending a personal therapy session. Keeping a clear structure helped the group focus on the project, with the research team maintaining realistic expectations regarding the potential impact for the project by highlighting that this was an opportunity for the project to help others, but it was unlikely to provide ‘the final answer’. We were reassured from the feedback from CYP that the opportunity to have their voices listened to and potentially impact change was an extremely positive experience. We present some practical suggestions based upon our own experiences for the involvement of PPIE in evidences within Table 3.

**Table 3:** Practical suggestions for meaningful PPIE in complex evidence syntheses

Practical suggestions for meaningful PPIE in complex evidence syntheses^a^
Consider the involvement of a dedicated PPIE facilitator. Provide clear and accessible information on the purpose of the evidence synthesis, how the synthesis will be carried out and how PPIE will be incorporated. Foster a shared motivation to co‐produce meaningful, accessible and useful findings. Maintain flexibility in approach to accommodate changing needs and demands on the PPIE group. Enable flexibility in methods of contribution, for example email, telephone and face to face. Maintain contact between meetings. Regularly check‐in with individuals to ensure that the involvement is mutually beneficial for them and the research. Maintain realistic expectations regarding the potential impact of PPIE contributions on the project. Provide opportunities for members of the PPIE group to take ownership of the work by taking part in the dissemination of findings, for example by attending conferences, making podcasts and writing blog posts and plain language summaries.

^a^ No formal evaluation of our PPIE was undertaken. These suggestions are based upon the reflections of the authors only.

## 10 CONTINUING REFLECTIONS

Following our experiences of working alongside CYP and their parents, the research team reflected on the challenges of maintaining engagement with the PPIE team across the 2‐year project life cycle and beyond. This way of working is typical for academics, but unusual to PPIE team members who are not being formally paid for their involvement. During this time, CYP and their parents had many competing demands on their time, including specific challenges of living with an LTC and mental health difficulties, school, work and maintaining relationships with friends and family.

Although we worked hard to adhere to the INVOLVE public involvement values and principles^17^ to meaningfully involve CYP throughout the project, we asked ourselves—could we have done more? What would a truly co‐produced evidence synthesis look like, and would this be a feasible and enjoyable way of working for all parties? True co‐production at this level would mean the establishment of a working relationship and sharing of research tasks prior to project conception, through grant application, conducting and writing up the project and dissemination. This would require training and for non‐researchers to engage with research tasks, which may not be particularly enjoyable, within a fast‐paced research environment. This may not be particularly appealing to young people and require a trade‐off between those who might have limited time and want to steer/speak from an informed position vs those who might have time for research tasks. Clear boundaries as to the responsibility of each stakeholder group would need to be established, and caution used to ensure people did not become overwhelmed by either the volume or content of the material they were working with. It is not clear, from our experience, whether CYP and their parents would welcome this level of responsibility. A blended model of co‐production and consultation may be more appropriate, to allow each stakeholder group to use their relevant skills and expertise to fulfil the demands of the project.

## 11 CONCLUSIONS

Through fully embracing the value of incorporating the views of CYP with lived experience of long‐term physical conditions and mental health issues within the project, we were able to identify clear opportunities for the views of the PPIE team to actively shape our research process, findings and dissemination strategy. In return, the CYP and their parents recognized the value of research and worked hard to maintain their involvement with the project. Our collaborative relationship was nurtured by a shared sense of respect and mutual desire to understand each other's experiences and needs, and deliver good quality research to help future CYP with physical long‐term conditions and mental health problems.

## CONFLICT OF INTEREST

All authors report no conflicts of interest to this work.

## Data Availability

Data sharing is not applicable to this article as no new data were created or analysed in this study.
